# Cutting to the Chase: How Matrix Metalloproteinase-2 Activity Controls Breast-Cancer-to-Bone Metastasis

**DOI:** 10.3390/cancers10060185

**Published:** 2018-06-05

**Authors:** Marilena Tauro, Conor C. Lynch

**Affiliations:** Department of Tumor Biology, H. Lee Moffitt Cancer Research Center and Institute, 12902 Magnolia Dr., Tampa, FL 33612, USA; marilena.tauro@moffitt.org

**Keywords:** bone, breast cancer, progression, MMP inhibitor, MMP-2, matrix metalloproteinase-2, bone metastasis, therapy

## Abstract

Bone metastatic breast cancer is currently incurable and will be evident in more than 70% of patients that succumb to the disease. Understanding the factors that contribute to the progression and metastasis of breast cancer can reveal therapeutic opportunities. Matrix metalloproteinases (MMPs) are a family of proteolytic enzymes whose role in cancer has been widely documented. They are capable of contributing to every step of the metastatic cascade, but enthusiasm for the use of MMP inhibition as a therapeutic approach has been dampened by the disappointing results of clinical trials conducted more than 20 years ago. Since the trials, our knowledge of MMP biology has expanded greatly. Combined with advances in the selective targeting of individual MMPs and the specific delivery of therapeutics to the tumor microenvironment, we may be on the verge of finally realizing the promise of MMP inhibition as a treatment strategy. Here, as a case in point, we focus specifically on MMP-2 as an example to show how it can contribute to each stage of breast-cancer-to-bone metastasis and also discuss novel approaches for the selective targeting of MMP-2 in the setting of the bone-cancer microenvironment.

## 1. Introduction

The vast majority of breast cancer patients that succumb to the disease do so because of metastatic burden. While breast cancer metastasizes to major organs such as the lung, brain, and liver, studies have shown that almost 70% of patients will have evidence of bone metastasis upon autopsy, making it a common occurrence in patients with advanced disease [1,2]. Bone metastatic breast cancer is currently incurable, and treatment plans typically focus on limiting cancer-associated bone destruction, chemo/radiotherapy, and palliative care, which aim to slow the cancer growth and enhance the patients’ quality of life. Understanding the mechanisms that facilitate breast cancer metastasis to bone, dormancy, awakening, and establishment can yield novel therapeutic targets to limit or prevent the manifestation of bone lesions.

The metastatic cascade paradigm illustrates how cancer cells must invade through the extracellular matrix (ECM) to intravasate into the surrounding blood supply and extravasate into the tissue of the metastatic site. The ability to process the ECM is a key tool used by cancer cells to negotiate these steps. Matrix metalloproteinases (MMPs), a family of 23 secreted and membrane-bound proteinases, have long been associated with intravasation and extravasation because of their ability to process the majority of ECM components [3]. However, broad-spectrum inhibitors targeting MMP activity failed in clinical trials, arguably in large part as a result of the fact that our understanding of MMP biology was limited. Subsequent studies have revealed distinct pro- and anti-tumor roles for individual MMP family members, suggesting that a more targeted approach would be efficacious. With the advent of new technologies that can deliver drug payloads specifically to the tumor microenvironment and improved chemistry approaches to enhance specificity, the selective inhibition of MMPs known to play causal roles in cancer progression is now a reality. The question then arises—which MMPs do we selectively target? In the context of breast cancer progression and bone metastasis, several MMPs have been shown to play important roles, and of these MMPs, MMP-2 has several noted roles (Table 1). Here we present MMP-2 as an example of how individual MMP family members contribute to the disease via the processing of matrix and non-matrix substrates and discuss the strategies that can be used to selectively target MMP-2 while sparing the activity of other MMP members.

## 2. MMP-2 Expression in Breast Cancer and Correlation with Metastasis-Free Survival

The MMPs are a 23-member family of zinc-dependent endopeptidases, mainly involved in the proteolytic degradation of the ECM [16]. They are also capable of processing non-matrix-related proteins such as cadherins, integrins, growth factors, cytokines, and cell surface receptors. Further, they have been shown to activate other MMPs and proteases [7,17]. As a consequence, MMPs are key regulators of cell–cell interaction and play important roles in a wide variety of normal biological processes, including embryonic development, morphogenesis, angiogenesis, ovulation, cervical dilation, bone tissue remodeling, wound healing, and apoptosis [18]. However, MMPs have been most widely studied in the context of pathological processes such as cardiovascular disease, arthritis, ischemia, and, most notably, cancer and metastasis [19]. In the tumor microenvironment, cancer cells express MMPs, but, importantly, MMP expression is often induced and localized to the surrounding stromal and immune cells [20,21].

**Figure 1:**
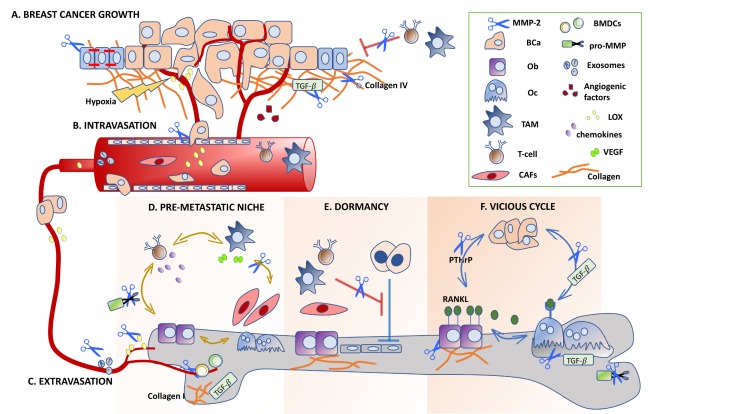
Roles for matrix metalloproteinase-2 (MMP-2) in the breast-to-bone metastatic cascade. (**A**) MMP-2 contributes to breast cancer growth and immune evasion by regulating the availability of growth factors, including TGFβ. (**B**,**C**) Extracellular matrix (ECM) degradation is critical for intravasation/extravasation into/out of the blood vessels, and MMP-2 is capable of processing several ECM components, including type I collagen, to facilitate these processes. (**D**) MMP-2 activity in the pre-metastatic niches of the bone promotes ECM remodeling of the niche and the recruitment of stromal and immune cells that in turn facilitate the recruitment of disseminated breast cancer cells. (**E**) New evidence shows that TGFβ isoforms are important mediators of dormancy entry/exit, and MMP-2 regulation of the TGFβ isoform bioavailability may play an important role in these processes. (**F**) MMP-2 is capable of controlling the activity and bioavailability of several growth factors important in the breast cancer cell, osteoblast, and osteoclast vicious cycle. Inset illustrates the major cellular players and factors involved. Breast cancer, Ob: osteoblasts; Oc: osteoclasts; TAM: tumor-associated macrophage; CAF: cancer-associated fibroblasts; BCa; BMDCs: bone-marrow-derived cells.

MMP-2 is a prime example of how individual MMP family members can contribute to disease progression by processing a vast repertoire of ECM and non-ECM substrates, which in turn raises questions regarding implications for each step of the metastatic cascade (Figure 1) [16,22,23,24].

In breast cancer, MMP-2 levels/expression have been shown to correlate with poorer overall survival. For example, mRNA levels of MMP-2 and its closely related MMP family member MMP-9 are significantly higher in MDA-MB-231 and MCF-7 breast cancer cell lines (MDA-MB-231 and MCF-7) compared to the normal HS578Bst cell line [25]. Further, low MMP-2 and MMP-9 mRNA levels are associated with better overall survival for breast cancer patients [25]. Supporting these findings, MMP-2 positivity appeared to be associated with an increased risk of death (1.8-fold higher) during the first 10 years of follow-up [26]. Furthermore, MMP-2 was studied together with clinic-pathological parameters (tumor size, histological type, nuclear and histological grade, stage, lymph node status, ER, and PR), tissue inhibitor metalloproteinase-2 (TIMP-2), Ki-67 score, and p53 mutational status and was found to be associated with more invasive phenotypes [27] and correlated with lymph node metastasis (positive vs negative: OR of 1.91; 95% CI of 1.17–3.12) [28].

While these studies show a correlation between MMP-2 and breast cancer aggressiveness, preclinical animal models have also defined causal roles for MMP-2 in promoting invasion and metastasis via ECM degradation and angiogenesis [29,30]. For example, overexpression of TIMP-2, an endogenous inhibitor of MMP-2, significantly decreased breast-to-brain metastasis, and it was shown that astrocyte-derived factors could regulate MMP-2 expression via ERK1/2 (extracellular signal–regulated kinases 1/2) signaling [31,32]. Amplified in breast cancer 1 (AIB1) can also regulate MMP-2 expression via the co-activation of PEA3 (polyoma enhancer activator protein) transcription factors that bind to the MMP-2 gene promoter. Using the MMTV (mouse mammary tumor virus)–polyoma middle T antigen (PyMT) mouse model of mammary tumorigenesis and metastasis, genetic ablation of AIB1 significantly impaired metastasis, with a noted reduction of MMP-2 expression and epithelial-to-mesenchymal transition (EMT) [33]. Similarly, using an intracardiac model of injection, overexpression of MMP-2 in MDA-MB-231 was shown to enhance the number and extent of detectable breast-to-bone metastases [6].

While clear functions for cancer-cell-derived MMP-2 are implicated in breast cancer progression and metastasis, stromal-derived MMP-2 has also been shown to play key roles in the disease [34]. Analysis of MMP-2 mRNA by in situ hybridization showed that fibroblasts are a major source of this specific MMP [35]. Recent studies have also defined roles for fibroblast MMP-2 contributing to the outgrowth of breast metastases in the lung. Ex vivo, wild-type lung fibroblasts significantly promoted the growth of breast cancer spheroids, an effect that was not observed with MMP-2-null fibroblasts. This absence of growth promotion observed in the MMP-2-null fibroblasts could be rescued via the addition of exogenous TGFβ, suggesting that MMP-2 control of TGFβ bioavailability was responsible [34]. In the context of bone metastasis, the growth of PyMT cell lines was significantly delayed in MMP-2-null mice compared to controls, with TGFβ bioavailability again implicated [36].

Taken together, these data suggest that MMP-2 activity is necessary for the successful metastasis of primary breast cancer cells to the secondary site. As discussed further on, evidence in the literature suggests that MMP-2 can contribute to several key components of the metastatic cascade, including cancer cell survival, proliferation, anoikis, immune invasion, angiogenesis, EMT, pre-metastatic niche development, intravasation, extravasation, dormancy, and outgrowth in the metastatic tissues due to its substrate repertoire.

## 3. MMP-2 Contribution to the Early Steps of Breast Cancer Progression and Invasion

In order to grow successfully, breast cancer cells must become resistant to apoptosis and proliferate, and MMP-2 has been implicated in these processes. In addition to roles in MMP-2 processing and the activation of latent TGFβ, MMP-2 can regulate the availability and activity of several other growth factors [36,37,38]. For example, MMP-2 processing growth factors, including heparin-binding EGF-like growth factor (HB-EGF), in response to gonadotrophin-releasing hormone (GnRH) activation can promote cell proliferation, with HB-EGF activity linked to breast cancer growth [39,40]. Neo-vascularization of the primary cancer via angiogenesis is critical for growth beyond 1 mm and is a hallmark of cancer progression [41]. Studies have previously shown that MMP-2 can contribute to angiogenesis by inducing vascular endothelial growth factor (VEGF) expression or processing factors important for regulating VEGF bioavailability, such as connective tissue growth factor (CTGF) [42,43]. MMP-2 can further facilitate angiogenesis of the tumor microenvironment by cleaving ECM molecules such as type IV collagen and coordinating with αvβ3 integrins [44]. Another hallmark of cancer is the evasion of immune-cell-mediated cytotoxicity, and MMP-2, via the regulation of chemokine activity, can also play a contributory role in this aspect of the disease. For example, several members of the chemokine ligand (CCL) family, such as CXCL12, are processed by MMP-2 into inactive forms that limit the activation of CXCR4-positive immune infiltrating cells [45]. MMP-2 has also been shown to be responsible for the shedding of the MHC class I polypeptide-related sequence A (MICA), which is important for stimulating natural killer (NK) and T-cell anti-tumor immunity [46]. Further, the interaction between MMP-2 and the toll-like receptor-2 (TLR2) on the surface of dendritic cells can increase OX40L expression and promote an anti-inflammatory response by inducing the type 2 polarization of T-cells [47].

The EMT program is a key component of the metastatic cascade, in which epithelial cells become mesenchymal-like and acquire migratory/invasive phenotypes [41]. The initiation of EMT results in enhanced MMP expression, including that of MMP-2 [48]. The increased expression of MMPs by cancer cells consequently facilitates their invasion through the underlying basement membrane. Slug, Snail and Twist have been documented as key mediators of the EMT program, but microenvironmental factors such as matrix stiffness can also contribute to epithelial cancer cell transition to a more mesenchymal state [49]. In breast cancer, increased deposition of collagen I and its cross-linking induces a nearly 10-fold stiffening of the mammary stroma [50]. Matrix stiffness in the primary breast tumor correlates with disease aggressiveness and regulates a variety of cellular processes, including cell proliferation, migration, and differentiation [24,51]. The expression of lysyl oxidase (LOX) by the breast cancer cells causes cross-linking of collagen fibers that in turn increases matrix tension. Paradoxically, increased ECM stiffness has been associated with increased invasiveness [52] and enhanced expression of proteases, including MMP-2. While roles for amoeboid/non-proteolytic ECM invasion have been described, MMP-2, in coordination with MMP-14, are key regulators of type I collagen degradation and cancer cell invasion through type I collagen encapsulating the growing breast cancer [53,54,55].

## 4. MMP-2 and Pre-Metastatic Niche Development

Steven Paget’s “seed and soil” hypothesis postulates that a receptive microenvironment (soil) is required for malignant cells (seeds) to engraft distant tissues and form metastases [56]. It has recently been recognized that pre-metastatic niches develop in distant organs long before seeding by metastatic cancer cells and that factors derived from the primary tumor are responsible. In this regard, LOX has also been shown to play a role in the formation of bone pre-metastatic niches. Hypoxia-inducible factor (HIF) regulates LOX expression and accumulation at sites of future metastasis in the bone, causing the cross-linking of collagen IV and the increased adhesion of CD11b+ myeloid cells that secrete proteases, including MMP-2 [57]. MMP-2, in turn, is responsible for generating collagen IV peptides as a byproduct of proteolytic activity—fragments with chemo-attractive properties for bone-marrow-derived cells and future metastatic breast cancer cells recruited to the pre-metastatic niche [58].

Recent focus has shifted to understanding the roles of exosomes in the genesis of the pre-metastatic niche [59]. These nanometer-sized vesicles contain a variety of proteins and RNAs that can be transferred between cells, leading to tumor microenvironment modulation. Fluorescently labeled B16 melanoma exosomes have been shown to accumulate at pre-metastatic sites in the lung and significantly enhance melanoma-to-lung metastasis via the transfer of exosomal mesenchymal-to-epithelial transition (MET) (a receptor of hepatocyte growth factor—HGF) to bone-marrow-derived cells in the lung parenchyma [60]. Exosome-derived factors, including heat shock protein 90, can also induce the expression of MMPs, such as MMP-2, in the pre-metastatic niche, with the resultant matrix remodeling contributing to niche formation [61]. Interestingly, analysis of the exosome content derived from cancer and stromal cells reveals the presence of membrane-bound and soluble MMPs, including MMP-2 [62]. The precise function of exosome-derived MMPs remains to be determined, but emerging studies have shown that MMP-14 can promote the activation of exosome pro-MMP-2 that in turn can enhance the remodeling of type I collagen, a major component of the bone ECM.

## 5. Dormancy and ECM Interaction

Breast cancer patients often develop recurrence in distant organs years to decades after the successful eradication of the primary tumor and adjuvant therapy [63]. Understanding the process of how disseminated breast tumor cells (DTCs) enter into the dormancy program and the conditions that drive awakening from the dormant state can reveal insights into how to prevent disease recurrence. DTCs, because of their quiescent state or low turnover, are thought to be protected from neoadjuvant or adjuvant therapies and immune surveillance. Fortunately, the mechanisms that enable cancer cells to evade these potential cytotoxicities are beginning to emerge. It is known that breast cancer cells that have extravasated and taken up residency in the endosteal or vascular niches of the bone can engage in a dormancy program (REF), and the factors that drive dormancy appear to be highly tissue- and isoform-specific [64]. For example, TGFβ has three isoforms, and TGFβ2 expressed in the bone marrow promotes dormancy in breast cancer via the activation of the TGFβ receptors. This leads to MAPK p38α/β activation, resulting in an ERK/p38 low signaling ratio with reductions in the cell-cycle promoter, CDK4, facilitating entry into dormancy [65]. MMPs, and in particular MMP-2, may also support tumor dormancy, releasing bioactive fragments of ECM that can inhibit angiogenesis, such as endostatin [66], restin [67], arrestin [68], and all three chains of type IV collagen [69]. Because of its role in regulating TGFβ availability, MMP-2 may also play an important role in activating the TGFβ2 isoform, thereby facilitating DTC entry into dormancy.

Mechanisms specific to cancer dormancy in the bone endosteal niche have been described in the context of prostate cancer, for which the binding of annexin II receptor expressing prostate cancer cells to osteoblasts promotes the expression and secretion of growth arrest-specific 6 (GAS6). GAS6 in turn binds to the receptor Axl and results in the suppression of prostate cancer cell growth [70]. Axl activation has been shown to induce the expression of several MMPs, including MMP-2, which could initiate the dormancy program via the activation of the TGFβ2 isoform, for example, or by the recruitment of other DTCs to pre-metastatic niches (REF). Similar mechanisms could potentially be employed by disseminated breast cancer cells. Leukemia inhibitory factor (LIF) receptor (LIFR) is expressed on DTCs that are dormant in bone. Ligands that bind to LIFR, such as the IL-6 family members, LIF, and oncostatin M, are generated by bone-lining osteoblasts. Of note is that the activation of LIFR has been shown to induce MMP-2 expression, which again may facilitate the migration of DTCs into endosteal niches and the subsequent entry into dormancy [71]. Interestingly, the loss of LIFR expression on breast cancer cells, hypothesized to be due to low oxygen conditions of the bone-marrow microenvironment, enables the awakening and establishment of active osteolytic lesions [72].

Mechanisms surrounding exit from the dormancy program are also being revealed. Interestingly, the TGFβ1 isoform, which is highly sequestered in the bone matrix, promotes the awakening of dormant cells, suggesting distinct roles for TGFβ isoforms in regulating dormancy entry/exit, and, by proxy, the proteases, such as MMP-2, that control TGFβ bioavailability [73]. In the vascular niche, the stable blood vessel architecture surrounding the DTCs assists in maintaining the dormancy state, but destabilization or turnover of the vessels requires the “sprouting” of endothelial cells, with the tip cells secreting the TGFβ1 isoform and periostin that awaken the dormant DTCs [73]. Again, MMP-2 is associated with neo-angiogenesis-controlling endothelial cell migration and the bioavailability of VEGF and TGFβ1 [43,74,75]. Interestingly, periostin has also been shown to promote MMP-2 expression [76]. In the endosteal niche, factors involved in mobilizing hematopoietic stem cells (HSCs) can perturb the DTCs occupying those niches, causing cancer cell outgrowth. Osteoclasts are essential for HSC mobilization, and osteoclast-derived MMP-9 cleaves kit-ligand, which is responsible for maintaining HSC quiescence. Osteoclast activity in the endosteal niche may contribute to the awakening of dormant cancer cells as a result of increased bone remodeling, with resultant high levels of bioavailable TGFβ1 isoform, generated in part by MMP-2, contributing to micrometastatic outgrowth [63,65]. While MMP-2 can regulate the activity and availability of factors that contribute to dormancy and awakening, it is likely that the spatial and temporal expression of the protease, in addition to the concentration of available substrates, ultimately determines MMP-2’s potential roles in these processes.

## 6. MMP-2 in the Vicious Cycle of Bone Metastasis

Once awoken or active, micro-metastases must cooperate with the bone microenvironment in order to establish and grow. While EMT plays a role in metastasis, metastatic cells reverse-engineer the process and undergo MET, which in turn allows for interaction with bone stromal cells [77,78]. For example, re-expression of the epithelial cell adhesion marker E-cadherin on breast cancer cells facilitates heterotypic binding to N-cadherin-expressing osteoblast cells in the osteogenic niche [79]. This interaction promotes the activation of mTOR and AKT pathways and results in the proliferation of the metastatic cancer cells. These studies indicate that the interaction between bone lining osteoblasts and arriving metastatic cancer cells are critical early interactions for the genesis of micro-metastases prior to osteoclast involvement. In bone, the reciprocal interactions between metastatic breast cancer cells and the bone stromal cells have been described as the “vicious cycle”. Breast cancer cells secrete a myriad of factors, such as parathyroid hormone-related protein (PTHrP). PTHrP is a 36 amino acid protein that binds and signals through the PTH1R G-protein coupled receptor, and its expression is directly correlated with the formation of osteolytic bone metastases [80]. Recently, MMPs, including MMP-2, have been reported to process PTHrP_1–36_ into smaller fragments, including PTHrP_1–17_, which dampens the ability of the hormone to induce osteolysis [81]. Interestingly, however, PTHrP_1–17_ retains the ability to promote osteoblast differentiation and bone formation by signaling via the protein kinase C (PKC) pathway [81]. The precise role for this novel MMP-generated product in other cellular components of the bone-cancer microenvironment remain to be determined.

In bone, PTHrP is a potent stimulator of the receptor activator of nuclear κB ligand (RANKL) in addition to other molecules such as prostaglandin E2 (PGE2) and interleukins [82,83,84]. RANKL, a member of the tumor necrosis factor (TNF) family, is a trimeric cell-surface ligand that is essential for promoting the fusion of myeloid cells into multinucleated osteoclasts [85]. Interestingly, RANKL is susceptible to cleavage by a number of MMPs, including MMP-3, -7, and -14 [9,86,87]. Differential effects for the soluble form of RANKL on osteoclast formation have been noted, with MMP-3 and -7 promoting osteoclast activation and MMP-14 inactivating RANKL. These differences may be dependent on the juxtamembrane cleavage site of RANKL. Once formed, osteoclasts bind to the surface of the mineralized bone matrix subsequent to osteoblast retraction and demineralize the bone via the release of hydrochloric acid [88]. Tight resorptive seals permit localized acidification under the osteoclast. Approximately 90% of the bone matrix is composed of type I collagen, and the osteoclast secretion of cathepsin K, an acidophilic type I collagenase, facilitates the breakdown of the collagen fibers [89]. MMP expression, including that of MMP-2, is also elevated at the tumor–bone interface in response to osteoclast activity [9,36,90]. Transcytosis of the bone matrix products through the osteoclasts in vesicles results in pH buffering prior to apical secretion [91,92]. The subsequent release of bioavailable growth factors in turn supports the growth of the breast cancer cells, thereby generating a feed-forward “vicious cycle” of tumor–bone interaction [93].

During bone apposition phases, osteoblasts not only lay down ECM proteins but also incorporate a number of growth factors and cytokines into bone, such as TGFβ, that can be released during osteoclast-mediated resorption [94,95,96]. Bone is the richest source of TGFβ in the body, which is synthesized as a homodimeric pro-protein that requires processing in order for activation [97]. The pro-peptide, known as the latency-associated protein (LAP), together with TGFβ, is further complexed to latent TGFβ binding proteins (LTBP1-4). LTBP3 is concentrated in the skeletal tissue and plays an important role in regulating TGFβ bioavailability [98,99]. MMP-2 has been shown to cleave LTBP3 and LAP, and therefore it is a key regulator of TGFβ bioavailability in bone. Paradoxically, MMP-2 is highly expressed by bone-forming osteoblasts, for which it plays a role in the remodeling of non-mineralized ECM, known as osteoid, and is important for canule formation during osteoblast terminal differentiation into osteocytes [100]. The initiation of the bone remodeling process involves retraction of osteoblasts from the mineralized bone matrix surface, a process that requires the degradation of the non-mineralized osteoid matrix. MMP-2, as a type I collagenase, plays a role in this process and in the liberation of sequestered TGFβ from the osteoid. Genetic ablation of host MMP-2 in mice, and hence also in bone-lining osteoblasts, significantly delayed the growth of bone metastatic breast cancer, with reduced levels of TGFβ indicated as a contributing mechanism.

MMP-2 secretion from other host cellular sources can also promote breast cancer growth. For example, leukocyte-derived MMP-2 and -9 are able to activate latent TGFβ residing in the ECM by proteolytic cleavage of LTBP1 and subsequently enhance breast cancer growth [101]. Conversely, TGFβ also has an important immunosuppressive role in promoting the recruitment of immune-suppressive myeloid cells [102,103]. MMP-2 may also play potential roles in regulating other factors that control bone stromal functions. For example, the fibroblast growth factor receptor 1 (FGFR1) activity is critical for osteoblast maturation and bone formation [104]. MMP-2 activity results in the shedding of a soluble FGFR1 ectodomain that retains the ability to bind FGFs and prevent FGFR1 signaling. This in turn could potentially impact osteoblast-mediated bone formation and exacerbate the osteolytic nature of the bone metastatic breast cancers [105].

## 7. MMP-2 Selective Inhibition in Bone

Given the prominent role for MMP-2 activity in breast cancer progression and metastasis, the question remains as to whether the therapeutic targeting this individual MMP family member is feasible. MMP-2 is ubiquitously expressed, raising the possibility of off-target effects or dose-associated toxicity as noted in previous MMP-inhibitor clinical trials [106]. To overcome this risk, numerous strategies for selective MMP inhibition or site-specific delivery of MMP inhibitors have been, or continue to be, identified [107].

Currently, bisphosphonates are widely used in the clinical setting to prevent cancer-induced bone disease and skeletal-related events such as pathological fracture [108]. Bisphosphonates bind to hydroxyapatite in the bone and cause osteoclast apoptosis upon resorption by inhibiting the mevalonate pathway [109,110]. Interestingly, bisphosphonates have been shown to inhibit MMP activity at high concentrations [111,112]. By chemically altering the bisphosphonate structure, a series of compounds were developed with significantly superior MMP inhibitory profiles, including to that of MMP-2. Using the MMP-2-specific reagents, it was recently shown that this approach can be used to treat bone metastatic breast cancer in pre-clinical animal models of the disease. The bisphosphonic moiety allows for the specific targeting of the MMP-2 inhibitor to the skeletal tissue and the site of bone metastasis. high-performance liquid chromatography (HPLC) analyses showed the accumulation of the bone-seeking MMP inhibitors (BMMPIs) in the skeletal tissue over time. Further, the use of MMP-2-selective near-infrared probes showed that MMP-2, as opposed to other MMPs expressed in the tumor–bone microenvironment such as MMP-13, were selectively inhibited by the administered BMMPIs [113]. These MMP-2-specific BMMPIs significantly reduced the growth of PyMT and 4T1 murine mammary cancer cell lines in bone by limiting TGFβ bioavailability compared to controls. The BMMPIs also significantly protected against cancer-induced bone resorption. Because bisphosphonates are well tolerated in the clinical setting, the translation of MMP-inhibitor-based bisphosphonates for the treatment of incurable bone metastatic breast cancer is highly feasible. Strategies to selectively target MMP-2 activity in other tissues or the delivery of cancer-specific payloads containing MMP-2 inhibitors may allow for the inhibition of this potent proteinase in other steps of the metastatic cascade.

## 8. Conclusions

Bone metastatic breast cancer is a common progression for many thousands of women with late-stage disease. Despite medical advances, the disease remains incurable. MMPs have for many years been associated with breast cancer progression and metastasis and often play causal roles in the process. As discussed, MMP-2 contributes to each step of the breast cancer metastatic cascade by virtue of its ability to cleave a large repertoire of matrix and non-matrix substrates. While enthusiasm for MMP inhibition was dampened by the broad-spectrum clinical trials conducted over 20 years ago, our knowledge of the mechanisms through which individual MMPs contribute or protect against cancer progression has increased profoundly. This, coupled with the advent of new chemical design strategies and drug-delivery systems, may allow for translational studies with novel MMP inhibitors in the near future.

## Figures and Tables

**Table 1:** Role of matrix metalloproteinases (MMPs) in bone metastatic breast cancer.

MMP	Enzyme	Substrate	Role in Breast Cancer to Bone Metastasis	Ref.
MMP-1	Collagenase-1	Collagens (I–III, VII, VIII, and X), gelatin, and MMP-2 and -9	Primary tumor growth and collagen cleavage	[4]
MMP-2	Gelatinase-A	Gelatin, collagens IV–VI and X, elastin, and fibronectin	Collagen cleavage, extracellular matrix (ECM) degradation, bioavailability of growth factors (TGF-β/PTHrP), regulation, angiogenesis, pro-MMP/cytokine activation, immune surveillance regulation, and bone pre-metastatic niche formation	[5,6,7]
MMP-3	Stromelysin-1	Collagens (III–V and IX); gelatin; aggrecan; laminin; elastin; plasminogen; MMP-2/TIMP-2; and MMP-7, -8, -9, and -13	Bone remodeling and ECM degradation	[8]
MMP-7	Matrilysin	Collagens (IV and X); gelatin; aggrecan; laminin; elastin; plasminogen; and MMP-1, -2, and -9	Primary tumor growth, osteoclast formation, and RANKL processing	[5,9]
MMP-9	Gelatinase-B	Collagens (IV, V, VII, X, and XIV), gelatin, aggrecan, elastin, plasminogen, MBP, and IL-1β	ECM processing, bone resorption and remodeling, regulation of VEGFA bioavailability, and angiogenesis promotion	[10,11]
MMP-13	Collagenase-3	Collagens (I–IV, IX, X, and XIV), gelatin, plasminogen, fibronectin, and MMP-9	Osteoblast morphology regulation and bone resorption, type I collagen processing, and physiologic bone development	[12,13]
MMP-14	MT1-MMP	Collagens (I–III); gelatin; fibronectin; vitronectin; proteoglycans; and MMP-2 and -13	Cell invasion, migration and metastases, pro-MMP2 activation, and type I collagen cleavage	[14,15]

TIMP: Tissue Inhibitor of Metalloproteinase-2; RANKL: Receptor activator of nuclear factor kappa-B ligand; MBP: myelin basic protein; MT1-MMP: Membrane-type 1 matrix metalloproteinases; IL: Interleukin.
